# Mine Waste Disasters on the Zambian Copperbelt: Regulatory and Community Concerns

**DOI:** 10.1007/s00267-026-02444-x

**Published:** 2026-04-09

**Authors:** Charles Poleni Mukumba, Makweti Sishekanu, Lochner Marais

**Affiliations:** 1 Centre for Development Support, University of the Free State, Bloemfontein, South Africa; 2 Sustainable Minerals Institute (SMI) of the University of Queensland, Brisbane, QLD Australia

**Keywords:** mine waste, tailings storage facility, disaster, GISTM (Global Industry Standard on Tailings Management), legislation, regulation

## Abstract

The accumulated waste produced by a mine can pose risks to communities in the vicinity. Three disasters occurring within 15 months on the Zambian Copperbelt have refocused attention on mine waste management and disasters in Africa. The paper investigates these three disasters in the light of the changing regulatory environment and liberalisation of the mining sector in Zambia. Although the country has been slow to adapt, some regulatory progress has been made in the past ten years. However, our interviews with two focus groups and twelve key informants and our assessment of media and government reports found little recognition of the need for co-development of disaster responses and risk reduction. Some progress has been made regarding transparency in the Global Industry Standard on Tailings Management (GISTM) and in Zambia’s regulations. But the enforcement of the regulations remains weak in Zambia. Old waste legacies and new value chains linked to liberalisation hold disaster risk. The result is livelihood disruption, environmental degradation, damage to health, and sometimes fatalities. We call for Zambia to be proactive and forge a closer link between the existing ecological regulations and disaster risk reduction, rather than relying on dealing with the consequences when a disaster happens.

## Introduction

The mining industry produces huge quantities of waste, varying between 95% and 99.9% of the material extracted (Nassar et al. 2022). This has been increasing over the past two or three decades as improved mining technologies allow for the mining of lower grades of ore (Calvo et al. 2016). Managing this waste poses both immediate and long-term risks for the environment and nearby communities. The increased likelihood of storage failures increases the need for risk transparency and disaster risk reduction.

Many African countries have liberalised their mining sector since the mid-1980s. There have been four generations of mining liberalisation: the liberalisation of markets and reduction of government control (late 1980s and early 1990s) (Hilbom and Green 2019); the introduction of more structured environmental regulations to ensure local benefit and human rights (late 1990s and early 2000) (Hilbom and Green 2019); an enhancement of stakeholder involvement (mid-2000s) (Hilson 2020); and an increase in transparency and accountability efforts (from 2010) (Besada and Martin 2015). Liberalisation has created much-needed foreign investment (Lin et al. 2022) and local benefits (Luning 2012), but African countries remain ill-prepared for the effects on environmental regulation and mine waste management and have struggled to ensure local benefits of mining (Owusu-Koranteng 2008). As mining technology improved and markets were liberalised, many companies began to mine lower grades of ore and re-mine existing mines. One result has been an increase in waste. The companies have had to deal not only with the new waste but also with the historical waste legacy. Furthermore, the liberalisation of the mining industry has created value chains that have benefited informal mining, thereby generating new waste.

Faced with this situation, the regulatory environment, struggling to keep up with global trends, is not coping with the increased production in liberalising contexts and seldom emphasises disaster risk reduction. Although only about 5% of reported tailings storage facility (TSF) failures between 1915 and 2021 occurred in Africa (Lin et al. 2022), this is likely to increase as the expanding mining sector and inadequate regulations increase the risks. The risk is aggravated by fluctuations in copper prices, as cycles of mining expansion and contraction shape investment decisions, ownership changes, and regulatory oversight. For example, the copper price boom of the mid-2000s, driven by rapid industrialisation in China, triggered turnover in ownership of the Zambian Copperbelt (Liebenthal and Cheelo 2020). The subsequent downturn after 2008 led to mine closures, retrenchments, deferred maintenance, and weakened oversight, heightening environmental and governance risks.

Between December 2023 and February 2025, three catastrophic incidents related to mine processing and waste occurred on the Zambian Copperbelt: a mudslide from an old waste dump (a pile of rock and soil which the mine did not consider contained economically recoverable minerals), spillage from a heap leach pond (a pond that collects and stores the chemical solutions used to extract metals from the ore), and the collapse of a TSF (a dam that stores the residue after the resources have been extracted). We use the word “waste” to refer to any waste product, including rock and soil, tailings, and chemicals used in processing. We assess these three cases against the historical and current regulatory environment. Our case studies indicate that regulators are struggling to develop comprehensive regulations and enforce environmental regulations, as well as incorporate broader ideas from disaster risk reduction studies into these regulations. Although many of these problems exist elsewhere, they are particularly acute in Africa, where liberalisation has not been met with appropriate waste management laws, environmental control, disaster risk reduction laws and enforcement. Of particular concern are the low levels of transparency about the disaster risk and, consequently, the minimal emphasis on disaster risk reduction. Failure to acknowledge the risk hampers risk reduction strategies. At the same time, we recognise the conflict between liberalising the mining economy and dealing with historical waste legacies. Learning from these African experiences is essential because African mining is crucial to the energy transition. Society will judge the mining industry by its safety and environmental protection record. Failure is often catastrophic for the environment and for land-connected communities.

## Literature Review

### Waste, TSFs, and Failures

The processing and storing of mine waste and tailings has long-term implications. Historically, standard ways to “dispose” of waste were dumps for unprocessed ore of little economic value and dams for tailings called TSFs (Adamo et al. 2021). As technology, production systems and value chains change, this leftover material that had little value originally can become valuable. Among the many concerns about the safety of waste dumps are instability (Amarsaikhan et al. 2018), the consequences of seismic activity (Wasilewski and Skotniczny 2015), environmental impacts (Zhao et al. 2012) and water pollution (Zhu et al. 2019).

Heap leach mining is a common way of extracting metals from lower-grade ores. This is a hydrometallurgical process in which a water-based chemical solution percolates through heaps of crushed ore to dissolve the targeted mineral, which is then recovered from the enriched solution. Approximately 21% of copper mines use this method (Franson 2017). Managing the chemical process, the stockpiles and the water (in ponds) is risky. The release of water containing sulphuric acid used for extracting copper is harmful to ecosystems (McBride et al. 2017). Risk factors include inadequate design of ponds and drainage systems, heavy rain, and stockpiles affecting water flows (Davies et al. 2000; Adamo et al. 2021).

Tailings are the finely ground waste materials left over after the minerals have been separated from the ore in a processing plant, using water based or chemical methods (Edwards 2019). Worldwide, there are about 3500 TSFs, with from two to five failures annually (Hudson-Edwards et al. 2024). Between 2015 and 2021 about 2650 people were killed by TSFs failures, about 317,000 had their livelihoods disrupted, and about 5000 km^2^ of land was contaminated (Hudson-Edwards et al. 2024). Common human causes of failure are construction and design faults, regulatory shortcomings and skimped maintenance (Hudson-Edwards et al. 2024). Non-human or physical causes include slope and foundation slippage or subsidence, extreme weather events and earthquakes (Kemp 2020). It is essential to understand the role played by regulatory factors in these disasters. The quality of regulations and compliance in Africa is a cause for concern (Kemp et al. 2021; Kemp et al. 2024). The earlier literature tends to focus on the physical causes of failure, but the more recent literature increasingly emphasises the human factors and the way the regulatory environment ignores them, particularly community culture, lack of transparency, and avoidance of disaster terminology.

The Expert Panel on the causes of the Brumadinho Dam I failure emphasised the role of company culture (Independent Consulting Committee for Investigation of the Brumadinho Tailings Dam (I) Failure. 2020). The Panel found that the mining company Vale SA commonly ignored warning signs and auditor findings. The one-dimensional focus on profits, driven by cost-cutting measures, compromised safety. Weak oversight and poor internal reporting compounded the problem. The board and managers seldom questioned reports and there was no culture of transparency. The result of the company’s poor governance, risk management practices and safety culture was poor design and poor maintenance.

Kemp et al. (2024) say the mining industry finds it hard to be transparent about TSF risk, particularly where weak governance and corruption are prominent. There is little proactive disclosure of disaster risks and several systematic barriers inhibiting transparency, like a lack of capacity, absence of procedure, reluctance to be transparent, the fact that many countries industry regulations do not require disclosure, and limited community agency (Kemp et al. 2024).

The mining industry tends to avoid disaster terminology. For example, it commonly refers to “failures” rather than “disasters”. Consequently, mines seldom develop disaster response plans. Disaster studies have experienced several changes since the heydays of positivism in the 1970s, which tended to view disasters as largely natural. Social constructivist thinking, as in the work by Oliver-Smith (1996; 1999) in the 1990s, has since changed these assumptions. One contribution of social constructivism has been to see disasters as socially embedded in human behaviour. The current trend is to integrate elements of positivism and social constructivism. In practice, this means giving equal prominence to the physical and social aspects in disasters.

Kemp et al. (2024) say the mining industry should learn lessons from five main themes of the disaster literature: the way disasters spring from social processes, the concept of vulnerability, understanding the root causes of a disaster, focusing on the temporal and spatial aspects, and looking beyond the technical reasons for a disaster. For the lawmakers, disasters highlight the need for a socio-legal view instead of the dominant doctrinal paradigms of law that underpin command-and-control regulation.

### The Global Industry Standard on Tailings Management (GISTM)

After the Brumadinho disaster in Brazil in 2019, the ICMM revised the standards for TSFs. The result was the GISTM in 2020 (ICMM 2020). The six topic areas and fifteen principles of the GISTM are shown in Table 1.

**Table 1:** GISTM topic areas and principles

Topic areas	Principles
I – Affected communities	1. Respect the rights of project-affected people and meaningfully engage them in decisions that affect them. 2. Develop and understand social, cultural, environmental and economic contexts. 3. Respect human rights, including the rights of Indigenous Peoples.
II – Integrated knowledge base	4. Develop an interdisciplinary knowledge base to support safe tailings management. 5. Establish and implement a robust, multi-disciplinary risk assessment and management framework.
III – Design, construction, operation	6. Develop and implement safe design, construction, operation, and closure practices. 7. Develop robust monitoring systems to track performance and address any deviations. 8. Establish and implement an effective governance system for tailings facilities.
IV – Public disclosure and access to information	9. Assign accountability at the highest organisational levels for tailings facility safety. 10. Establish and implement governance structures to support safety and integrity. 11. Ensure financial and human resources are available for tailings safety across the facility lifecycle.
V – Emergency response and long-term recovery	12. Develop, test, and maintain an effective emergency response plan. 13. Prepare for long-term recovery in the event of a failure.
VI – Public disclosure	14. Ensure public disclosure of information about tailings facilities that is accessible, understandable, and appropriate. 15. Report against standardised global indicators for tailings facilities.

Source: ICMM 2020

Although the GISTM relates specifically to storage facilities for tailings (one kind of mine waste), its basic principles are more widely applicable to storage of all kinds of mine waste, and are thus applicable to all three case studies in this paper. In addition to the 15 principles, five overarching aspects of the GISTM are relevant to this paper. The first is accountability and the principle of zero harm (related to principles 1 and 3). Company must prioritise safety and communicate it to their employees. The second is a stronger community and stakeholder engagement (related to principles 2 and 9–16). The GISTM requires meaningful engagement with affected communities throughout the lifecycle of a TSF. Mining companies must provide transparent and publicly available information. For example, they must stipulate geolocations and risk assessments for every facility. Mines must co-develop these risk assessments with nearby communities. Risk assessment must include free, prior and informed consent. The third is the integration of environmental and social considerations, including human rights considerations (related to principles 2, 3 and 5). The fourth is the risk-informed approach (related to principle 6). All TSFs must undergo a risk assessment, and there must be independent reviews, external assurance and transparency. The fifth is emergency preparedness and response and recovery plans (related to principles 12 and 13).

For Hopkins and Kemp (2021), the GISTM has not gone far enough because some decisions were made through compromising processes. They believe there could have been stricter technical requirements for upstream TSFs, disposal methods, consequence management, financial liability, rights, preventive infrastructure and non-direct but long-term impacts. There are also concerns about standards for governance and controls during implementation. The final version has been criticised for downplaying the social aspects of disasters. Despite the objective of zero harm, there is no explicit focus on having no tolerance for harm. Finally, it was feared that implementation of the GISTM might be hindered by the inherent power relationships between mining companies, government and local communities

### Liberalising Mining in Africa

#### Background

Policies after independence in African countries tended to curtail capital-intensive mining. Many post-independence regimes nationalised mining. Economic growth rates after independence were generally lower than expected. Several of these countries had budget deficits and could not pay their debt. The debt crisis led to Structural Adjustment Programmes, with conditionality clauses that included liberalising these economies (Mlambo et al. 2021).

Despite the liberalisation, two main concerns remain: the regulatory aspect and the local benefit. This paper is mainly about the regulatory aspect. Adapting the environmental legislation to the liberalising environment has been difficult. Liberalisation reintroduced global mining companies and created informal links to existing mining value chains. In addition to mining companies from the North, Chinese mining companies have become prominent. Access to critical African minerals is central to China’s development (Kohnert 2024) and to broader geopolitical interests. Significant Chinese investments have been made in Zambia and the DRC (Mlambo et al. 2021). This restructuring has implications for the African states, prompting discussions about dependency, exploitation and sustainable development

**Fig. 1:**
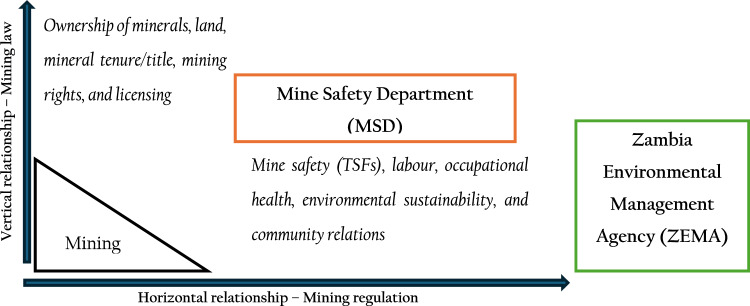
Distinction between mining *law* and mining *regulation*. Source: Ndulo 2013, [41]

We borrow from Ndulo (2013) who distinguished between mining *law* and mining *regulation*, as shown in Fig. 1. Mining law (the vertical relationship with communities) defines ownership of minerals (and mineral rights), mineral tenure, the licensing regimes, and the acquisition of exploration and mining rights; while mining regulation (the horizontal relationship with communities) defines the ordering and monitoring of day-to-day operational functions, including mine safety and environmental sustainability, labour, occupational health, and community relations.

#### Post-colonial first-generation mining statutes: 1964–1990

After independence, mining law in Zambia vested the ownership of minerals and title in the state and the President, on behalf of the Republic. The Mines Acquisition (Special Provisions) Act No. 28 of 1970 laid the foundation for nationalisation (Government of Zambia 1970). Nationalisation had to break with colonial practice and link political independence with economic benefit. State control of mining operations through the Zambia Consolidated Copper Mines (ZCCM) became the norm (Oshionebo 2021). Consequently, post-colonial mining legislation was statist, state-centred, and underpinned by the authority over ownership and control of minerals. There was strict control over foreign investment, and the regulatory responsibility shifted to the state-owned companies. The state regulated its mining operations as a lawmaker and a regulator.

An overemphasis on the vertical aspects (law) downplayed the horizontal aspects (regulation) during nationalisation. For example, in Part V of the Mining Regulations of 1973, the state had to ensure that mines provided a mine plan (Government of Zambia 1973). The Chief Inspector of Mines had to inspect the plans before commencement. The mine manager had to align the day-to-day operations of the mine with the plan (Government of Zambia 1973). For example, a surface plan had to show the position of any principal surface erection, including TSFs (Government of Zambia 1973, regulation 202(1)). Consequently, the mine plan had a regulatory framework, with the mine manager having to enforce the mine plan. The surface plans included designing, managing and monitoring TSFs and dumps.

The regulations had to prevent a collapse that might endanger people or property. However, the mining companies had discretion regarding enforcement. The discretionary purview of this regulation lay in the “practicable reasonability” of managing the tailings and other surface constructs. Although there was a reference to damage to property, the regulations did not specifically refer to damage to the environment.

While mining companies had to enforce the regulations through a mine manager, they also had to satisfy the government’s inspector (Government of Zambia 1973, regulation 605). The inspector had to notify the mine manager of the absence or presence of any mining matter deemed dangerous, defective, injurious or a threat to health, and anything that could cause property damage (Government of Zambia 1973, regulation 301(2)). According to Regulation 302(1), the inspector could compel the mine manager to remedy defects. Furthermore, the inspector had to report offences for possible prosecution to the Director of Public Prosecutions. If convicted, offenders were punishable by a fine or imprisonment or both (Government of Zambia 1973, regulation 305).

#### The economic liberalisation era, second-generation statutes: 1991–2000

By 1990, the mining sector was stagnant. Consequently, the Zambian Government liberalised the mining economy by promulgating private sector-oriented mining laws and deregulating mining operations. The IMF and the World Bank aided this shift (Oshionebo 2021). According to the World Bank (1992), liberalisation had to balance three aspects: the profitability of private investors, the revenue of the government, and environmental protection. Furthermore, the government had to provide a conducive, deregulated environment for the private sector, reduce investment risks, guarantee investment protection, and eliminate unwarranted government interventions. The government’s role in the mining sector was deregulation and withdrawal (Oshionebo 2021).

Consequently, the Zambian Government promulgated two statutes: the Environmental Protection and Pollution Control Act of 1990 (EPPCA) (Government of Zambia 1990), and Chapter 213 of the Laws of Zambia, the Mines and Minerals Act No.31, 1995 (Government of Zambia 1995).

##### The EPPCA (1990) (repealed)

In response to widespread environmental concerns, the Zambian Government enacted the EPPCA in 1990 (Lindahl 2014). The Copperbelt region had over 10,000 hectares of land covered by mineral waste (World Bank 2002). The EPPCA established the Environmental Council of Zambia (ECZ). The EPPCA included references to water, air, waste, pesticides and toxic substances, ionising radiation, and natural resources. However, the Act did not relate the environmental issues to mining. This neglect is probably because mine safety and tailings were already subject to the Mines and Minerals Act. By default, this drew a *de facto* boundary for the ECZ’s environmental regulatory mandate.

The ECZ’s statutory mandate was advisory, although some functions related to waste were controlling pollution, conducting research, assessing impacts, requesting information, and publicising information. In some cases, the new mining regulations excluded the new mines from historical environmental legacies. For example, the Mines and Minerals (Environmental) (Exemptions) Order SI No. 19 of 2000 (Schedule 1, paragraph 2) used such an exception instrument for Konkola Copper Mines (KCM) PLC (Government of Zambia 2000). Historical legacies created under previous regimes could become disaster risks in the new dispensation.

The law requires the ECZ to establish a Standing Technical Advisory Committee, constituted of experts in the field. Relevant fields of expertise included the environment, pollution, pesticides, toxic substances, noise, ionising radiation, hazardous wastes, and waste management (Government of Zambia 2000). Regarding waste, *Section twenty-three* stipulated the responsibilities of the Council to establish water and quality standards and pollution control standards, and conduct investigations (Government of Zambia 2000). Despite this Section’s value, there were four problems: the advisory nature or mandate of the regulator, a focus on when spills occur (as opposed to a preventive approach, both in design and in relation to communities), the exemption of some historical legacies, and the many confidentiality clauses. For example, the ECZ could not publicise relevant information on TSFs, tailings storage design, engineering, and management by mining companies, as this was a Mines Safety Department (MSD) jurisdiction. The Environmental Management Act, 2011, eventually repealed the EPPCA, 1990, in 2011.

##### Chapter 213, The Mines and Minerals Act, 1995 (repealed)

Chapter 213 of Zambia’s Mines and Minerals Act of 1995 introduces Mining Development Agreements (MDAs) to attract and protect mining investments (Government of Zambia 1995). MDAs allowed the Minister to grant mining licences with legally binding terms. Clause 23(2) gives large-scale licence holders exclusive rights to mine and manage operations, including TSFs, but subject to environmental protection obligations outlined in an ecological plan (Government of Zambia 1995). This plan would address pollution prevention, waste treatment and land and water protection, and minimise adverse environmental effects. Through clause’s 77(1), the government retained oversight through the Director of Mine Safety, who had to ensure compliance with environmental conditions (Government of Zambia 1995). In case of non-compliance or failure, the Director could take remedial actions and charge the costs to the licence holder under a polluter-pays principle. The Zambian Government established the Environmental Protection Fund to support the enforcement of this principle. Mining companies had to deposit funds to cover potential environmental rehabilitation costs upon licence expiry or termination, for non-compliance with ecological plans, or for debts owed to the Republic following remedial actions by the state. However, this only applied at the end of mining. The coordination between environmental regulators and mining authorities remained weak. Public opposition led to the repeal of MDAs under the Mines and Minerals Development Act No. 7 of 2008.

##### Statutory Instrument No.28, Environmental Impact Assessment Regulations of 1997 (in force)

Under Section 6 and 96 of the EPPCA, 1990, the Environmental Impact Assessment (EIA) Regulations require project developers to prepare an Environmental Project Brief (EPB) EIA before implementation (Government of Zambia 1997a). Regulations 3 and 7 make EPBs and EIAs mandatory. TSFs, waste dumps and processing plants (including heap leach ponds) all fall within the definition of a “project” and require authorisation. These provisions closed avenues for avoiding EIA obligations, subjecting quarrying and open-cast mining to the Second Schedule’s EPB requirement (Government of Zambia 1997a). At the same time, mineral processing (including heap leaching), ore reduction, smelting, and refining require a full EIA. Companies must submit EIAs to the environmental regulator, not the Minister under the Mines and Minerals Act, 1995. The ECZ thus gained indirect authority to regulate the mining processing, waste (including tailings and waste dumps) from the planning stage, applying precautionary and preventive principles. Post-assessment audits had to monitor compliance. These requirements were controversial as developers had to oversee themselves, creating discretionary leeway (Sishekanu and Katati 2021). Nonetheless, the EIA Regulations establish a legally binding foundation for mining developments. Cancelling an EIA approval cancels the associated mining licence. However, the Council’s Standing Technical Advisory Committee lack of mining engineering expertise to assess complex projects and the design of TSFs hampered effective implementation.

##### The Mines and Minerals (Environmental) Regulations, Statutory Instrument No.29 of 1997 (in force)

The Regulations require mining developers to submit an EPB and Environmental Impact Statement (EIS) to the Director of Mines Safety before prospecting, exploring or commencing mining operations (Government of Zambia 1997b). A mine had to appoint a mine manager with at least two years experience in mining environmental management. The Director reviews EPBs and brings mining EIAs under ecological law to the ECZ (Government of Zambia 1997b).

An EIS must include maps of TSFs, waste, dumps, the mining right area, tonnage and chemical composition of dumped material and a rehabilitation plan (this would include heaps from heap leaching). The mine must update the EIS annually, and independent auditors must audit compliance. Before dumping waste or placing it in a TSF, developers must notify the Director, specifying material, site description, classification, TSF design and safety precautions. The Fourth Schedule gives the required technical data, including scaled plans, design records, boundaries, and construction details, emphasising that TSF safety hinges on engineering design. While safety provisions focus on pre-approval, day-to-day management depends on regulations 12–17, which rely on self-regulation. Developers must create operational rules, appoint a competent inspector for weekly checks, monitor drainage, and submit periodic reports to the Director (Government of Zambia 1997b).

This approach is an example of a second-generation statute’s deregulatory ethos, where government oversight depends on five assumptions: the integrity of engineering design of TSFs; adequacy of the environmental management plan; thoroughness of updates and reports; robustness of EIS audits; and independence of external auditors. The first and third are engineering matters, while the second and fourth relate to environmental safeguards. The fifth fills the gap between public regulation and corporate self-monitoring. Yet this still undervalues the social aspects of disasters.

#### Post-liberal, development-oriented, third-generation statutes, 2000 to present day

When the Mines and Minerals Development Act (2008) (Government of Zambia 2008) repealed the Mines and Minerals Act, 1995, abolishing Mining Development Agreements (MDAs), this marked a subtle return to nationalistic policies, framed as resource nationalism and indigenisation framed as resource nationalism and indigenization with an orientation towards social protection. These statutes emphasise socially responsible mineral exploitation. In *Nyasulu*, Justice a condemned a mining company for actions that deprived communities of constitutional rights. This shift signalled a new paradigm in mining’s “horizontal” relationship with local communities. Two Acts define this third-generation framework: the Environmental Management Act (EMA) No. 12 of 2011 (amended by Act No. 8 of 2023) (Government of Zambia 2023) and the Minerals Regulation Commission Act No. 14 of 2024 (Government of Zambia 2024).

The EMA strengthens environmental rights, public participation and access to information. It enables legal action where spills from TSFs or other mining activities breach the right to a safe, clean environment, supplementing agency enforcement. The EMA shifts enforcement from mine safety to environmental law when contamination occurs, making tailings and waste management a statutory and common-law obligation. The Environmental Management Act gives the Zambia Environmental Management Agency (ZEMA) a statutory duty to review EIAs and cancel EIA approvals for non-compliance. In practice, the cancellation of an EIA could result in the revocation of the mining licence. Amendments made in 2023 broaden EIA definitions, require registered experts, and introduce continuous compliance monitoring.

The MRC Act formalises the Minerals Regulation Commission to bridge gaps between legal principles and community protection. Section 6 mandates regulation and monitoring of mining operations, collaboration with ZEMA, and auditing of environmental management, including progressive rehabilitation and mine closure (Government of Zambia 2024). There is a focus on safety, health and environmental protection, embedding precautionary safeguards into licencing. In negligence, the license conditions establish the standard duty of care. However, as with earlier regimes, the real weakness lies in enforcing compliance with licence conditions. The Act constrains public interest litigation by imposing limitation periods on legal actions, which is problematic for disasters with long-term impacts (for example, health).

## Methods

### Overview

**Fig. 2:**
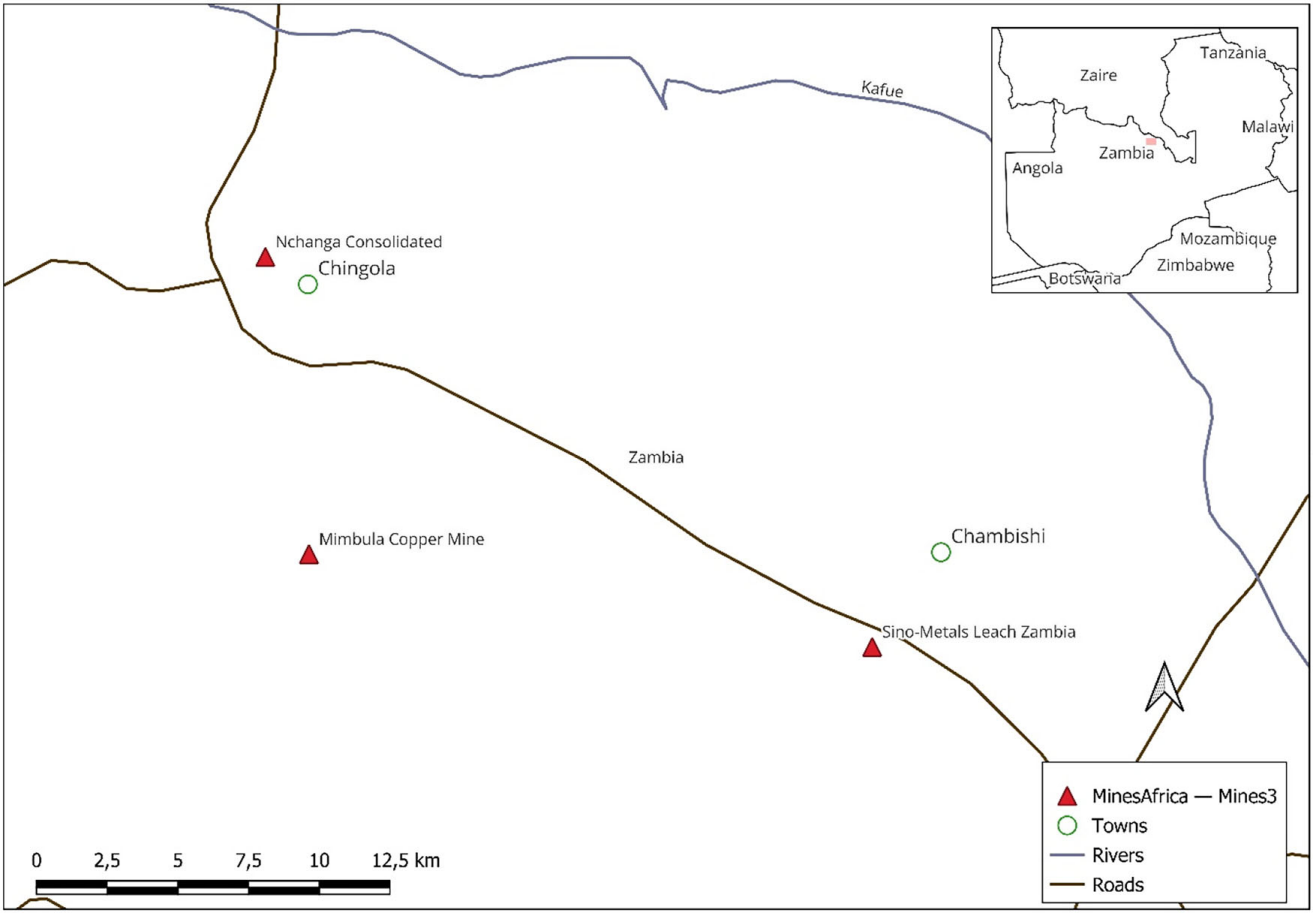
Location of mines in Zambia where accidents occurred

The paper is based on case studies of three disasters near Chingola, a mining town in Zambia’s Copperbelt Province (see Fig. 2), in the space of 15 months. We assessed the relevant mining and environmental legislation, regulations, media reports, official government reports, and responses. We collected primary data from participants about two of the disasters, at the Nchanga and Mimbula mines. We had access only to media and government reports to explain the failure of the Sino-Metals Leach Zambia TSF at Chambishi. For Nchanga and Mimbula we obtained data from two focus group discussions with ten participants and semi-structured interviews with twelve key informants, selected by convenience and snowballing sampling. We started by recruiting participants for the focus groups. These initial participants referred us to key informants who fitted our study’s criteria. This sequential approach enabled us to recruit participants directly affected by the mine disasters. The key informants included four former miners, three wives of miners, four farmers, and one local community leader. The four farmers owned a parcel of land along the Chabanyama stream, the source of water for their farms.

### Analysis

We used thematic analysis to analyse our primary interview data. We used Braun and Clarke’s (2006) phased framework. Interview and focus group transcripts were first read repeatedly to achieve data familiarisation. Initial coding was conducted inductively, allowing codes to emerge from the data rather than being imposed a priori. These open codes captured recurrent patterns related to regulatory practices, disaster experiences, livelihood impacts and perceptions of accountability. In a second stage, codes were reviewed, refined and grouped into broader categories through an iterative process of comparison across cases and data sources. This involved moving back and forth between the data, emerging themes and relevant literature on mine waste, disaster risk reduction and governance. While the analysis was primarily inductive, sensitising concepts from disaster studies and regulatory scholarship informed interpretation at later stages. Themes were finalised once saturation was reached and no substantively new codes emerged. Triangulation across interviews, focus groups and documentary sources strengthened analytical robustness.

We reviewed mining and environmental legislation, government reports, regulatory responses and media articles using a structured documentary analysis approach. Legal and regulatory documents were purposively sampled to include all statutes, statutory instruments and policy frameworks governing mine waste, tailings storage facilities, environmental protection and disaster management in Zambia from independence to 2025. We identified government reports and official responses through parliamentary records, regulatory agency websites and ministerial statements related to each disaster event.

Documents were analysed using a deductive–inductive framework. We derived deductive categories from the literature on mine waste governance and disaster risk reduction and included regulatory responsibility, enforcement mechanisms, transparency, accountability, and disaster preparedness and recovery. Inductively, additional themes emerged from close reading, including ambiguity over institutional mandates, avoidance of disaster terminology, and the treatment of historical waste legacies. For each document, we coded for the waste involved, causality, the regulatory action taken, and the presence or absence of risk disclosure and community engagement.

### Case Study Description

Global metal mining has expanded dramatically in recent decades and has become a major driver of environmental change through land disruption, water and air impacts, and biodiversity loss (Giljum et al. 2025). The renaissance of the Zambian Copperbelt is closely related to this expansion. Historically, copper was important for post-Second World War reconstruction (grid expansions, automobiles, weapons). More recently, it has become crucial in the development of renewable energy systems. Historically, it has had significant importance, supplying a significant share of global copper and remaining central to contemporary value chains linked to electrification, renewable energy, and the global energy transition. Its copper production underpins Zambia’s economy and foreign exchange earnings, while simultaneously embedding the region deeply within global extractive markets and geopolitical competition over critical minerals.

Chingola is a medium-sized mining city with a population of over 200,000, shaped by successive waves of labour migration linked to copper. The city exhibits high levels of informality, particularly in peri-urban and river-adjacent areas. Informal housing, informal water connections and artisanal livelihood activities are common. Informal settlements are often near mine infrastructure, tailings dams and waste disposal sites, reflecting both land availability and the historical prioritisation of production over environmental safety. Chambishi, located near the Sino-Metals site, is considerably smaller and more mono-functional. Its settlement pattern is tightly coupled to mining activities, with limited alternative employment opportunities and fewer formal urban services. Although town-level data for Chingola and Chambishi are limited, UN-Habitat (2026) estimates that 70% of Zambia’s urban population lives in informal settlements. This high level of informality underscores the prevalence of informal housing arrangements in urban areas of the Copperbelt and provides context for the housing dynamics observed in the case studies.

Both towns originated as company-built settlements. This legacy remains relevant for understanding contemporary exposure to environmental risk. Company planning historically emphasised proximity between residential areas and mining operations to support labour efficiency, embedding communities within landscapes shaped by mine waste, polluted waterways and industrial infrastructure.

The miombo woodland, interspersed with grasslands and riparian vegetation along rivers and streams, dominates the landscape. These landscapes support small-scale agriculture, grazing and fuelwood collection. Consequently, local livelihoods are vulnerable to contamination from mining-related pollution. Livelihoods in both areas extend beyond formal mine employment and include small-scale farming, gardening, fishing, informal trading and artisanal mining. These activities depend on local environmental resources, such as water from streams in the Kafue catchment. Seasonal rainfall patterns intensify reliance on these resources and increase vulnerability to pollution events, flooding and tailings failures. Vegetation in affected areas supports subsistence agriculture and grazing. Consequently, contamination has direct implications for food security and household incomes. High levels of informality in settlement, livelihoods and resource use intersect with mining-related environmental hazards. This locational reality amplifies the social and ecological impacts of pollution incidents and shapes community vulnerability.

Although no specific data exists on artisanal mining in Chingola or the Copperbelt, Artisanal and Small-Scale Mining (ASM) is a common feature of Zambia’s mining landscape. Artisanal mining provides livelihoods to many thousands of households and contributes to multiple mineral sub-sectors, including copper, manganese, gold and gemstones. The ASM Database on Artisinalmining.org lists more than 400 small-scale miners and half a million miners. This is about a third of the estimated miners in SADC (Delve 2020).

### Limitations

This study has several limitations that should be acknowledged. First, the research adopts a qualitative case study design focused on three mine-waste disasters on the Zambian Copperbelt. While this approach allows for in-depth contextual and regulatory analysis, the findings are not intended to be statistically generalisable to all mining contexts in Zambia or elsewhere. Rather, they offer analytical insights that may be transferable to similar liberalising mining environments. Second, primary data collection relied on a relatively small sample of participants, drawn through convenience and snowball sampling. Although this approach was necessary to access affected community members and knowledgeable informants, it may have introduced selection bias, as participants with stronger grievances or experiences of harm may have been more likely to participate. The absence of interviews with mining company representatives and regulators further limits the ability to triangulate perspectives across all key stakeholder groups. Third, data availability varied across the three cases. For the Sino-Metals TSF failure, the analysis depended exclusively on media reports and official government statements, which may be incomplete or politically mediated. This constrained the depth of comparative analysis across cases. Finally, the study relies on retrospective accounts of disaster events, which may be affected by recall bias. Despite these limitations, the combination of documentary analysis, interviews and focus groups provides a robust basis for examining regulatory gaps, transparency challenges and disaster risk reduction in the Zambian mining sector.

### Ethics

Ethics approval was obtained from the General Human Research Ethics Committee at the University of the Free State UFS-HSD2024/2407. Participants provided informed consent.

## Three Mine Waste Disasters in Zambia

The three disasters were a mudslide from a waste dump at Nchanga Consolidated Copper Mine’s on 1 December 2023, a leach pond failure at Mimbula Copper Mine on 19 January 2025, and a collapse of the TSF at Sino-Metals Leach Zambia on 18 February 2025.

### Nchanga Consolidated Copper Mine

Nchanga Consolidated Copper Mine is a cobalt and copper-producing mine in Chingola. Underground mining started in 1937, and open-cast mining in 1952. The Zambian Government obtained a 51% share in the mine in 1970. The first large-scale waste dumps originated in the late 1950s. Today, the mining operations comprise an underground mine, a primary mine and nine satellite open pits, a refractory, ore stockpiles and TSFs. In March 2000, Anglo American (an ICMM member) acquired the Nchanga Copper Mine as mining in Zambia liberalised (Sikamo et al. 2016). In 2002, Anglo American withdrew, citing operating difficulties, low metal prices and the cost of ageing infrastructure (Musonda and Larmer 2023). Vedanta Resources PLC (an Indian and UK-based company, but not a member of the ICMM) acquired the mine in 2004.

Vedanta encountered challenges from the government, and from communities who accused it of negligence in its duty in a UK court (Lopez and Crosser 2019). The parties settled out of court, without providing details or Vedanta accepting liability. In 2019, the government charged Vedanta with failing to invest and evading taxes. The government seized the assets and put the mine in provisional liquidation (Lopez and Crosser 2019). This action damaged the economy and the socio-economic well-being of nearby communities. In September 2024, Vedanta and the government resolved the conflict. Vedanta agreed to recapitalise the mine with USD 1.3 billion and to produce more than 300,000 tons of copper annually.

On 1 December 2023, a mudslide occurred after heavy rains at Senseli Open Pit Mine where non-licensed artisanal miners had been mining a slag dump. We could not determine when the dump was established, but it most probably originates from before independence. Informal mining is common at Zambia’s copper mining sites, where artisanal miners work without proper safety procedures. These illegal miners usually enter mines without the knowledge of the licence holders and sell their copper to small smelting operations that provide copper to the global value chains – often linked to China.

The mudslide killed 26 people. The immediate response from the mine and the government was to initiate search parties at the disaster scene. GroundUp reported that the Zambian Government is revisiting its approach to artisanal mining, while the mine and the government are accusing one another of security lapses (Mafa and Subeti 2025a). There are allegations of the involvement of politically connected people (Mafa and Subeti 2025b) and child labour (Mafa and Subeti 2025a).

### Mimbula Copper Mine

The Mimbula Copper mine is located on the outskirts of Chingola. Between 1952 and 1982, the Nchanga Consolidated Copper Mines, owned by Anglo American, operated the mine. Between 1982 and 1999, following nationalisation, the ZCCM managed the mines. From 2000 to 2002, Anglo American took over, followed by Vedanta Resources and Zambia Consolidated Copper Mines-Investment Holdings (2002–2013). Currently the large-scale mining licence, granted in 2017, is held by Mimbula Minerals Limited, a Zambian subsidiary of Moxico Resources PLC.

In 2014, the Zambia Environmental Management Agency approved Konkola Copper Mine’s EIS to reopen the Mimbula II Open Pit Project. The mine started a heap leach and solvent extraction process in 2022. Heap leaching has been used in Zambia since the 1970s, but has become prominent in the past two decades (Solei and Tinkler 2016). Heap leaching allows for the mining of lower grades of ore because it reduces the cost of milling and smelting. Heap leach ponds usually contain a water-based solution of chemicals (sulfuric acid for copper) that dissolve metals from the ore. Consequently, the operator must manage the ponds, as water spillage will harm the environment.

On 19 January 2025, the emergency heap leach pond embankment weakened and the pond’s contents spilt into the Chabanyama stream (Government of the Republic of Zambia, Parliament 2025). The acid effluent spillage affected 164 hectares, but no spill volume has been confirmed (Lusakatimes.com 2025). The affected residents had their crop fields, fish ponds and environments destroyed and polluted. The spillage eventually found its way into the Mushishima stream. The damage was exacerbated by a lack of lime dosing, suggesting little disaster preparedness by the mine There was also air pollution.

The government launched an independent investigation and a committed to keeping the mining company accountable (Lusakatimes.com 2025). It issued a compliance order, which will be lifted once the water is free of heavy metals (Lusakatimes.com 2025). The lack of basic and verifiable information about the leak might hamper legal processes.

### Sino-Metals Leach Zambia

Copper Mining at Chambishi by the Roan Selection Trust started in 1963 and the mine was nationalised by the Zambian Government in the 1970s. Initially, this was an open-pit mine, but underground mining began in 1974. The first TSF was created in the early 1970s, most likely to deal with the increased underground mining production. The mine closed in 1987. Liberalisation resulted in China Non-Ferrous Corporation Limited acquiring an 85% stake in 1998 and reopening the mine. Sino-Metals Leach Zambia, a China Non-Ferrous Corporation Limited subsidiary, now operates the mine, which consists of an open-pit copper mine, a concentrator plant, waste rock dumps, and a TSF. Increased waste requires new TSFs, but since 2019 Sino-Metals had raised the walls of the old Musakashi TSF, instead of building a new dam (UNDRR 2025).

TSF design and management practices have evolved significantly over time. Global statistics show that cumulative failure rates have declined as engineering standards and monitoring technologies have improved (Rana et al. 2022). TSF safety has improved through shifts to more stable construction methods (less upstream construction), stronger geotechnical design standards, enhanced water management, real-time monitoring technologies and stricter governance, meaning that modern failures are far more strongly associated with legacy dams than with contemporary best practice (Petley 2013; Rana et al. 2022). However, the TSF disaster examined in this study refers to a legacy TSF (i.e., raising the walls of an existing TSF) from a period when there was less emphasis on long-term stability and environmental risk. Although the Global Tailings Portal (2026) did not have TSF data for the failed TSF, four of the nine facilities listed from Zambia originated before 1991, and eight were upstream facilities.

Technically, the failure resulted from internal erosion through the wall separating the upper compartments (Petley 2025). On 18 February 2025, the dam burst, releasing 50 million litres of water containing heavy metals into the Mwambashi and Kafue rivers. The polluted water poisoned irrigation, drinking water and aquatic life (Ministry of Water and Sanitation 2025). Following the catastrophic collapse, the Mines Safety Department in the Ministry of Mines suspended the mine’s operations (Ministry of Water and Sanitation 2025) and issued a compliance order for the management of TSFs on 31 March 2025 (Mines Safety Department 2025). The order emphasises the poor management of waste materials (Mines Safety Department 2025). It expresses concerns about the poor design and construction of TSFs, gaps in the management of the TSFs, and poor monitoring and operational practices (Mines Safety Department 2025). The government fined Sino-Metals ZMW 1.5 million, which they duly paid and compensated the affected households (Business & Human Rights Resource Centre 2025).

## **Community Responses**

As noted earlier, we were able to interview the community only in regard to the incidents at the Nchanga and Mimbula mines. We identified three main concerns: the government’s inability to regulate mines, the consequences for livelihoods and health, and the role of artisanal and illegal mining.

### Government Regulations

Respondents commonly cited the weak enforcement of environmental regulations and insufficient institutional capacity. They said the government never penalises mining firms for their pollution. They had five concerns: the inactive environmental response linked to the liberalising policies and possible corruption, how common these disasters have become in the absence of mitigation mechanisms, the inherent conflict between mining and other economic activities, the lack of transparency, and the lack of disaster recovery plans.

Earlier, we outlined the regulatory problems associated with the ZEMA. The respondents felt that ZEMA is largely toothless. This ineffectiveness triggered speculation that its inaction results from corruption. Two respondents reacted as follows:

The ZEMA is there, but just on paper. You find that the rivers are polluted, and aquatic life is destroyed. And because of corruption, instead of doing inspections, they remain in offices and are given envelopes and go.

Where is the environmental agency? Where are they? Why are they quiet? Why are they mute? Could there be some big fish seated alongside or supporting such vices? Nobody knows. And we are still demanding answers.

The comment about being “given envelopes” implies corruption in the form of bribes. The liberalising mining policies since the 1990s are to blame. The fact that environmental structures only provide advisory services adds to the worry.

Respondents said catastrophic failures have become common. They compared the operations of the privatised mines to those when the government ran the mines. They said the government-run mine took law enforcement and pollution seriously. One respondent said:

Sad, sad. I am shocked to hear of the levels and frequency of water pollution. Because during the time of NCCM, ZCCM and partly KCM Anglo, it was a huge transgression. Even just to drop a trickle of acid in the natural water bodies. Arrangements were made in advance so that lime would be stationed where the effluent of the mining activities was being discharged. You know, this bridge, which is after the roadblock along the Chingola-Solwezi road, where there is a stream. All the time, there would be heaps of lime right there in case there was a spill over. The acidic effluent would find the lime to neutralise the pollution.

The lack of precautionary measures points to the Zambian government’s failure to include risk reduction and recovery plans in its disaster thinking. Respondents commonly questioned the adverse effects of mining on other industries, particularly agriculture. The primary concern was that the government is not planning the coexistence of mining and other industries. A typical remark was:

How do you mix agriculture, industry [mining] and habitation? People cannot live where they are smelting or washing copper, preparing, or whatever they call it. Have you seen that? It’s not right.

Many were concerned about the lack of transparency about the TSF disaster, with the full extent of the disaster not being disclosed (U.S. Embassy in Zambia 2025). Yet these concerns about transparency were voiced only after the disaster. Although we did not do interviews before the disaster, nobody we interviewed said the mine had warned them of a possible disaster. Furthermore, we found no evidence of transparency from the mine associated with the initial risk, as required by the GISTM and, to some degree, by current legislation.

Respondents complained that recovery plans were absent, suggesting little consideration for disaster thinking. They expressed frustration at the non-responsive government departments and mines when there was spillage from the Mimbula heap leach pond. They said there is no place to report a pollution incident, and no one would listen to their complaints. One respondent said:

No one pays attention whenever we experience water pollution from the mining activities. Nobody, nobody. I mean, because there is no one to report it to. To whom do you have to report it? There is no department. There is no employee. Nobody, nobody.

Despite legislation holding companies responsible, there is no proper response plan. There is no plan that would involve local people in the response. An appropriate management of disaster approach requires that mines and governments have prevention and recovery mechanisms. A simple reporting system seems an obvious necessity. The fact that the respondents say nobody is available points to the absence of recovery mechanisms.

### Livelihoods and Health

Although no person died in the pond or the TSF failure cases, there were concerns about the consequences for people’s livelihoods and health. Our respondents in the pond failure case described the damage to livelihoods. Two respondents described how the poisoned water made the soil unusable for agriculture and killed fish:

You see, water pollution caused by mining activities brings hunger. Once the rivers and our environment are polluted, hunger comes in because the field in which we are supposed to grow our crops is contaminated. The water becomes unsuitable for watering the crops. So, the whole community will be affected, as there will be a food shortage. It is not only the field that is concerned, but humans are also affected. Both humans and nature are concerned.

I was doing fish farming along the stream. I had two fish ponds, and it was nearing harvest time. All my fish are dead. The acid pollution from the mine instantly destroyed everything. Now I have no other source of capital to start another business. I also can t begin farming fish now. I have to wait until the water is good for the fish.

Hunger and lack of drinking water were major concerns. There were also health impacts on local communities after the TSF collapsed. In June 2025, the American embassy in Lusaka issued a warning citing “acute heavy metal poisoning” in the Mwambashi River and the Kafue River ecosystem. Since the incident, there have been public reports of widespread illness consistent with acute heavy metal poisoning (U.S. Embassy in Zambia 2025). In August, the embassy issued a health alert, saying new information had shown that “the extent of hazardous and carcinogenic substances” is beyond original estimates (U.S. Embassy in Zambia 2025). However, the Zambian government dismissed the claims, saying that the laboratory results showed that pH levels had returned to normal and that the water was safe (Drizit Zambia 2025).

A lawsuit has been filed on behalf of residents regarding health issues (Petley 2025). Those include respiratory problems, stomach pain, diarrhoea, rashes, eye irritation and coughing. The materials have dangerous levels of pollutants posing significant long-term health risks, including organ damage, congenital disabilities and cancer (Zadeh 2025). Most of the 449 farmers affected have dug traditional wells to access uncontaminated water for drinking and irrigation. However, the wells are shallow and already drying up with the onset of the dry season. The farmers whose fields are along the Chambishi stream have tried replanting crops without success due to acidic, waterlogged soils.

### **Illegal Mining Activities**

The Nchanga case involved the illegal mining of an old mining dump. A combination of market liberalisation, many unemployed people with mining skills, and a market value chain, primarily linked to China, increased informal mining. Most respondents said the young people of Chingola have resorted to illegal mining in response to not finding formal work, most of them are breadwinners who look after their families and aged parents. One respondent said:

They are unlawful miners because young men have to survive. Now, how do they survive? They are watching their parents, and they cannot support them. So, they are going to do illegal mining activities. Any metal they find is money. The young men are ready to die to feed their families.

The youth have few alternatives. Our respondents viewed the small Chinese smelting companies around Chingola town as having created a market for the illegal artisanal miners. They said the Chinese firms were “the biggest buyers” of the concentrates from these miners. One respondent said:

The illegal miners have their bosses. They do not just dig for themselves. So, when they get the material, their bosses get that stuff. Their bosses are the ones who take the material to the Chinese.

The respondents said youths participate in illegal mining because they have financial backers who sponsor their illicit mining activities, collecting and selling the ore to small Chinese smelting firms. They alleged that government officials were sponsoring the youths who are involved in illegal mining. One respondent said:

You will find that the ministers and councillors are the ones who are even at the forefront of illegal mining. They are the ones who are employing the boys. They are the chief buyers.

Nevertheless, most respondents said the illegal miners contribute positively to their communities.

## Discussion

Our study identifies five factors underlying these Zambian disasters that require a more detailed understanding in the aim of disaster risk reduction: liberalisation, inadequate regulations, historical legacies and ownership transfers, improved technology, and the avoidance of disaster terminology.

Liberalising the sector in the early 1990s was a logical economic response to a dormant mining industry. Historically, legislation gave colonial mining companies access to mining. Mining regulations followed later, but environmental thinking stagnated from the 1970s because the state both mined and regulated the mining. Waste management regulations were outdated when liberalisation occurred in the 1990s. To disrupt this path, during liberalisation, was not easy. The main goal was to revitalise mining, waste, and disaster regulations, which had been slow to catch up. Another consequence was the government’s limited ability to enforce the rules. For example, in none of these three cases was there any evidence of adherence to engineering standards, transparency regarding risk, or engagement with communities about possible disaster effects and responses. This is despite the requirements outlined in the GISTM and certain regulations.

The regulatory environment has some serious shortcomings. This goes deeper than a lack of enforcement. It raises the question of how the regulations could ensure more transparency and the co-development of disaster responses. The lack of openness and preparedness in the pond and TSF failure cases is indicative of a tendency towards a lack of transparency and disaster risk reduction in the Copperbelt. On the one hand, regulation requires a less regulatory emphasis and a focus on social legal aspects, seeking ways to facilitate risk reduction rather than imposing punitive measures in the event of a disaster. On the other hand, the lack of enforcement is a major problem. Furthermore, the diverse nature of the three accidents also highlights the difficulty in effectively dealing with different types of waste. Although the regulations provide broad provisions for this problem, it is in the implementation of the regulations that problems are likely to arise. Although the GISTM is a self-regulatory system introduced by the mining sector, there is very little indication in the behaviour of mining companies or the government that the basic principles are carefully implemented and regulated. The fact that none of these companies are ICMM members and are therefore not bound by the GISTM is, of course, the main reason. The GISTM’s focus on tailing does not help with the broader aspects of the three cases. Yet the current Zambian regulations make little reference to the co-development of disaster risk plans between communities and mining companies. Despite the limitations of the GISTM, Zambia can further develop the basic principles and apply them to a broader range of waste and operational procedures.

Ownership transfer and dealing with historical legacies were prime causes of all three disasters. All three cases saw a rapid turnover of owners. In two of the three cases, the re-use of an old TSF and an old waste dump, the legacies were prominent in the disasters. As noted earlier, in some cases, after liberalisation, new mining companies were exempted from responsibility for the historical legacies. This exemption attracted new mining investments. Under normal conditions, new mines and regulations must address these historical legacies and assess their associated risks, which may be costly and could necessitate security measures at old storage facilities. Exemption was, therefore, a direct attempt to promote mining at the expense of safety. Marais et al. (2024) noted that the failure to understand the historical context of mining activities was largely responsible for the disaster at the TSF in Jagersfontein. Historical risks require a deliberate assessment. An appropriate environment and disaster management response requires a historical understanding of old assets to determine current risks.

Improved technology has implications that extend beyond enhancing production. It makes it possible, and economically viable, to mine lower-grade ore and the ore in waste dumps from which previous technologies did not extract all the minerals or metals. The acceleration of mining activities results in more waste. In essence, improved technology creates more waste or destabilises existing waste through re-mining. The liberalisation of markets has increased market demand for ore from small-scale producers. The collapse of the old waste dump at Nchanga, where small-scale producers mined copper for these smelters, killed 26 people. From an environmental and safety perspective, this increase in waste requires appropriate regulations, which should include technical specifications for managing waste

Disaster terminology and disaster risk reduction are not given sufficient prominence. The Zambian Government’s regulations and the mining company’s responses to the cases in this paper would not even comply with some of its minimum standards in the GISTM. Overall, there is little transparency about TSF, ponds and waste dumps. We refer to three examples of where acknowledging disaster could be helpful. First, although mines are required to reveal the location of TSFs and waste dumps, this forms part of the mining licence process. Complying with the basic principle of risk transparency could be a good starting point. Second, although existing regulations require transparency regarding the existence or approval of new facilities, the enforcement of these issues appears problematic. Furthermore, the self-regulatory nature of the process with limited government oversight remains a problem in EIAs. Thirdly, disaster management and recovery plans are not a requirement. Our interview data portrays members of the affected communities in two of our case studies as merely victims of the disasters, without any preventive capacity – essentially because they were not involved in understanding risk and developing disaster response and recovery plans. The Zambian regulations urgently need to emphasise disaster terminology in managing TSFs, waste and operational risks. The main focus is on responding to disasters when they occur and then holding companies accountable. Effective risk reduction requires a greater emphasis on prevention and preparedness.

The increased metal mining requires regulatory responses (Giljum et al. 2025). The interface of disasters with artisanal mining, liberalisation, historical assets, and mine waste calls for a much more careful consideration in regulation and law enforcement. Despite the Zambian government’s regulations for addressing these issues, the coexistence and co-management of the risks will remain longstanding problems.

## Conclusion

The Zambian Copperbelt developed during the colonial era, when mining legislation ensured access for large international mining companies. When the Zambian government nationalised mining in the 1970s, it became both the regulator and operator of the mines. This dual role of the government contributed to the stagnation of environmental regulations. As the mining sector began to liberalise in the early 1990s, the primary objective was to streamline the business process. The emphasis was not on updating the environmental regulations. In fact, to ensure new investment, the mining agreements bypassed certain measures (for example, those dealing with historical waste legacies), and private sector mining companies had never been subject to regulation. Since the early liberalisation attempts, environmental regulations have attempted to catch up, but enforcing them has been difficult. The introduction of the GISTM, although only applicable to ICMM members, and despite some remaining shortcomings, does provide a framework for improved transparency and linking mining accidents (specifically, in its case, TSF failures) to disasters.

The evidence from the three disasters described in this paper indicates that mining disasters have been a common occurrence in Zambia. They occurred within 15 months, and our respondents have hinted that such disasters are more common than reported. Some of the causes are related to historical issues (adding to old waste dumps, expanding existing TSFs), and there may be many technical reasons for these failures. At the same time, the inadequacy of the regulatory environment (including enforcement) has contributed to these disasters.

More problematic has been the inability to improve transparency and the disaster risk reduction process in the mining industry. There is little evidence that mines have conducted community-informed risk assessments of their TSFs, old legacies, and other production processes, despite the GISTM. As a result, we found no evidence of disaster response or mitigation plans, nor any indication of co-development of such plans with local communities. Continuing the status quo ignores the value that social constructivism brought to a positivist-dominated disaster management process. Although environmental regulations require greater transparency, they are, in the main, silent on the link to disaster risk reduction.

This inability to link the regulations more carefully to disaster risk reduction is critical for two reasons. First, the worldwide demand for crucial metals and minerals will increase these risks in Zambia, where regulatory environments are weak. Secondly, this inability is likely to increase the number of disasters and their consequences for the environment and human life, simply because in Zambia the mining industry and governments view the failures as technical and mine-related. We call for a much closer link between existing environmental regulations and disaster risk reduction rather than dealing only with the consequences.

## Data Availability

Data is available on request.

## Appendix

**Annexure A: Mining and Environmental Legislation and Regulations Reviewed**

National legislation and regulations (Zambia)

• Mines Acquisition (Special Provisions) Act No. 28 of 1970

• Environmental Protection and Pollution Control Act (EPPCA), 1990 (repealed)

• Mines and Minerals Act No. 31 of 1995 (repealed)

• Environmental Impact Assessment Regulations, Statutory Instrument No. 28 of 1997

• Mines and Minerals (Environmental) Regulations, Statutory Instrument No. 29 of 1997

• Mines and Minerals (Environmental) (Exemptions) Order, Statutory Instrument No. 19 of 2000

• Mines and Minerals Development Act No. 7 of 2008

• Environmental Management Act No. 12 of 2011 (as amended by Act No. 8 of 2023)

• Minerals Regulation Commission Act No. 14 of 2024

International/industry standards

• Global Industry Standard on Tailings Management (ICMM 2020)

**Annexure B: Official Government Reports and Regulatory Responses**

• Ministerial Statements to Parliament on mining-related pollution incidents (2025)

• Mines Safety Department compliance orders related to TSF management (2025)

• Zambia Environmental Management Agency (ZEMA) EIA approvals and compliance notices

• Ministry of Water Development and Sanitation public statements following pollution events

• UNDRR briefing note on the Chambishi TSF collapse (2025)

**Annexure C: Media Reports**

• Business & Human Rights Resource Centre (2025) Zambia (2025). Residents sue China Nonferrous Mining over tailings spill, allege unsafe cleanup, surveillance and intimidation. Available at: https://www.business-humanrights.org/en/latest-news/1-zambia-residents-sue-china-nonferrous-mining-over-tailings-spill-allege-unsafe-cleanup-surveillance-and-intimidation

• Drizit Zambia (2025) Drizit Zambia responds to false allegations regarding Sino-Metals tailings disaster. Available at: https://drizit.com/wp-content/uploads/2025/09/29-08-2025-Drizit-Media-Statement-Z.pdf

• GroundUp

• Mafa, C. & Subeti, M. (2025a) *Child miners risk death in Zambia’s copper pits*. GroundUp. Available at: https://groundup.org.za/article/child-miners-risk-death-in-zambias-copper-pits/

• Mafa, C. & Subeti, B. (2025b) *The Zambian minister, the mysterious businesswoman and illegal mining*. GroundUp. Available at: https://groundup.org.za/article/the-zambian-minister-mysterious-businesswoman-illegal-mining

• Lusaka Times (2025) *Government to launch independent investigation into mining pollution of Kafue and Mwambashi Rivers*, 27 February. Available at: https://www.lusakatimes.com/2025/02/27/government-to-launch-independent-investigation-into-mining-pollution-of-kafue-and-mwambashi-rivers/

• U.S. Embassy in Zambia (2025) *Health alert – Acute heavy metal poisoning*. Lusaka: U.S. Embassy. Available at: https://zm.usembassy.gov/health-alert-acute-heavy-metal-poisoning/

• United Nations Office for Disaster Risk Reduction (UNDRR) (2025) *Collapse in Chambishi – Tailings failure is a canary in Zambia’s Copperbelt*. Available at: https://www.undrr.org

• Petley, D. (2025) *The 18 February 2025 Tailing Storage Facility Failure at Chambishi in Zambia*. EOS, 24 February 2025. Available at: https://eos.org/thelandslideblog/chambishi-tsf-1

## References

[CR1] Adamo N, Al-Ansari N, Sissakian V, Laue J, Knutsson S (2021) Dam safety. The question of tailings dams. Earth Sci Geotech Eng 11(1):1–26. 10.47260/egge/1111

[CR2] Amarsaikhan T, Shimada H, Wahyudi S, Sasaoka T, Hamanaka A (2018) Optimization of dump bench configuration to improve waste dump capacity of Narynsukhait open pit coal mine. Int J Geosci 9(6):379–396. 10.4236/ijg.2018.96024

[CR3] Besada H, Martin P (2015) Mining codes in Africa: emergence of a ‘fourth’ generation? Camb Rev Int Aff 28(2):263–282. 10.1080/09557571.2013.840823

[CR6] Braun V, Clarke V (2006) Using thematic analysis in psychology. Qualitative Res Psychol 3(2):77–101

[CR4] Business & Human Rights Resource Centre (2025) Zambia (2025) Residents sue China Nonferrous Mining over tailings spill, allege unsafe cleanup, surveillance and intimidation. Available at: https://www.business-humanrights.org/en/latest-news/1-zambia-residents-sue-china-nonferrous-mining-over-tailings-spill-allege-unsafe-cleanup-surveillance-and-intimidation

[CR5] Calvo G, Mudd GM, Valero A, Valero A (2016) Decreasing ore grades in global metallic mining, a theoretical issue or a global reality? Resources 5(4):36

[CR7] Davies M, Martin T, Lighthall P (2000) Mine tailings dams. When things go wrong. Las Vegas, Association of State Dam Safety Officials

[CR8] Delve (2020) State of the artisanal and small-scale mining sector. Washington, DC, Delve

[CR9] Drizit Zambia (2025) Drizit Zambia responds to false allegations regarding Sino-Metals tailings disaster Available at: https://drizit.com/wp-content/uploads/2025/09/29-08-2025-Drizit-Media-Statement-Z.pdf

[CR10] Edwards S (2019) Woeful tails of mining. Déjà vu in Minas Gerais, Brazil PreventionWeb. Available at: https://www.preventionweb.net/drr-community-voices/woeful-tails-mining-deja-vu-minas-gerais-brazil

[CR11] Franson J (2017) Cyanide poisoning of a Cooper’s hawk (Accipiter cooperii). J Vet Diagnostic Investig 29(2):258–260. 10.1177/1040638716687604 10.1177/1040638716687604 28114852

[CR12] Giljum S, Maus V, Sonter L, Luckeneder S, Werner T, Lutter S, Gershenzon J, Cole MJ, Siqueira-Gay J, Bebbington A (2025) Metal mining is a global driver of environmental change. Nat Rev Earth Environ 6:441–455

[CR13] Global Tailings Portal (2026) Tailings storage facility database, GRID-Arendal/Investor Mining and Tailings Safety Initiative. Available at: https://globaltailingsportal.org/

[CR14] Government of the Republic of Zambia, Parliament (2025) Ministerial Statement delivered by Minister of Water Development and Sanitation on the Pollution of Water Bodies caused by Illegal Mining Activities in the Country Lusaka: GRZ Parliament. Available at: https://www.parliament.gov.zm/node/12200

[CR15] Government of Zambia (1970) The Mines Acquisition (Special Provisions) Act No. 28 of 1970. Chapter 218. Lusaka: Government of Zambia

[CR68] Government of Zambia (1973) Mines and Minerals (Environmental) Regulations, 1973. Lusaka: Government Printer

[CR16] Government of Zambia (1990) The Environmental Protection and Pollution Control Act. Chapter 204 of the Laws of Zambia. Lusaka: Government of Zambia

[CR17] Government of Zambia (1995) The Mines and Minerals Act, No. 31 of 1995. Chapter 213 of the Laws of Zambia. Lusaka: Government of Zambia

[CR18] Government of Zambia (1997a) Environmental Protection and Pollution Control (Environmental Impact Assessment) Regulations. Statutory Instrument No. 28 of 1997, Regulation 2. Lusaka, Government of Zambia

[CR19] Government of Zambia (1997b) The Mines and Minerals (Environmental) Regulations. Statutory Instrument No. 29 of 1997. Lusaka, Government of Zambia

[CR20] Government of Zambia (2000) Mines and Minerals (Environmental) (Exemption) Order. Lusaka, Government of Zambia

[CR21] Government of Zambia (2008) Mines and Minerals Development Act, No. 7 of 2008. Lusaka, Government of Zambia

[CR22] Government of Zambia (2023) Environmental Management Act (EMA) No. 12 of 2011, as amended by Act No. 8 of 2023. Lusaka, Government of Zambia

[CR23] Government of Zambia (2024) Minerals Regulation Commission Act, No. 14 of 2024. Lusaka, Government of Zambia

[CR24] Hillbom E, Green E (2019) Period of deregulation 1985–2005. In: Hillbom E, Green E (eds) An Economic History of Development in Sub-Saharan Africa. Economic Transformations and Political Changes. Cham, Palgrave Macmillan, pp. 195–235

[CR25] Hilson G (2020) The Africa Mining Vision. A manifesto for more inclusive extractive industry-led development? Can J Dev Stud 41(3):417–431. 10.1080/02255189.2020.1821352

[CR26] Hopkins A Kemp D (2021) Credibility crisis. Brumadinho and the Politics of Mining Industry Reform. Sydney, Wolters Kluwer

[CR27] Hudson-Edwards K, Kemp D, Torres-Cruz L, Macklin M, Brewer P, Owen J et al. (2024) Tailings storage facilities, failures and disaster risk. Nat Rev Earth Environ 5(9):612–630. 10.1038/s43017-024-00576-4

[CR28] ICMM – International Council on Mining and Metals (2020) Global Industry Standard on Tailings Management (GISTM). London, ICMM. Available at: https://www.icmm.com/en-gb/our-principles/tailings/global-industry-standard-on-tailings-management

[CR29] Independent Consulting Committee for Investigation of the Brumadinho Tailings Dam (I) Failure, Extraordinary (CIAE-A) (2020) Executive Summary of the Independent Investigation Report – Vale S.A., requested by its Board of Directors, 20 February 2020, Vale S.A., Rio de Janeiro. Available at: https://vale.com/documents/d/guest/20-02-20_ciaea_report_i-1

[CR30] Kemp D (2020) Lessons for mining from international disaster research. In: Global Tailings Review (ed) A compendium of papers for the Global Tailings Review. London, Global Tailings Review, pp. 1–14

[CR31] Kemp D, Owen J, Lèbre É (2021) Tailings facility failures in the global mining industry. Will a ‘transparency turn’ drive change? Bus Strategy Environ 30(1):122–134. 10.1002/bse.2613

[CR32] Kemp D, Sharma V, Harris J, Blitz N, Williams D (2024) Disclosure hesitancy and disaster risk. A survey of tailings professionals in the global mining industry. Miner Eng 215: 108821. 10.1016/j.mineng.2024.108821

[CR33] Kohnert D (2024) Prospects and challenges for the export of rare earths from sub-Saharan Africa to the EU. Int J Afr Stud 4(1):87–110

[CR34] Liebenthal R, Cheelo C (2020) The boom–bust cycle of global copper prices, structural change, and industrial development in Zambia. In: Page J, Tarp F (eds) Mining for Change: Natural Resources and Industry in Africa. Oxford, Oxford University Press, pp. 374–396

[CR35] Lin S, Wang G, Liu W, Zhao B, Shen Y, Wang M et al. (2022) Regional distribution and causes of global mine tailings dam failures. Metals 12(6):905. 10.3390/met12060905

[CR36] Lindahl J (2014) Environmental impacts of mining in Zambia. Towards better environmental management and sustainable exploitation of mineral resources. Uppsala, Geological Survey of Sweden. SGU Report. Available at: https://www.resources.sgu.se/produketr

[CR37] Lopez C, Crosser M (2019) The UK Supreme Court considers whether Parent company Vedanta has a duty of care and may be held legally responsible for the harm caused by its Zambian subsidiary. https://opiniojuris.org/2019/01/22/the-uk-supreme-court-considers-whether-parent-company-vedanta-has-a-duty-of-care-and-so-may-be-held-legally-responsible-for-the-harm-caused-by-its-zambian-subsidiary/

[CR38] Luning S (2012) Processing promises of gold. A minefield of company–community relations in Burkina Faso. Afr Today 58(3):23–39. 10.2979/africatoday.58.3.23

[CR39] Lusakatimes (2025) Government to launch independent investigation into mining pollution of Kafue and Mwambashi Rivers, 27 February. Available at: https://www.lusakatimes.com/2025/02/27/government-to-launch-independent-investigation-into-mining-pollution-of-kafue-and-mwambashi-rivers/

[CR40] Mafa C, Subeti M (2025a) Child miners risk death in Zambia’s copper pits, GroundUp. Available at: https://groundup.org.za/article/child-miners-risk-death-in-zambias-copper-pits/

[CR41] Mafa C, Subeti B (2025b) The Zambian minister, the mysterious businesswoman and illegal mining GroundUp. Available at: https://groundup.org.za/article/the-zambian-minister-mysterious-businesswoman-illegal-mining

[CR42] Marais L, Kemp D, van der Watt P, Matebesi S, Cloete J, Harris J, Li Ern MA, Owen J (2024) The catastrophic failure of the Jagersfontein tailings dam. An industrial disaster 150 years in the making. Int J Disaster Risk Reduct 109: 104585. 10.1016/j.ijdrr.2024.104585

[CR43] McBride D, Ilankoon I, Neethling S, Gebhardt J, Cross M (2017) Preferential flow behaviour in unsaturated packed beds and heaps. Incorporating into a CFD model. Hydrometallurgy 171:402–411. 10.1016/j.hydromet.2017.06.008

[CR44] Mine Safety Department (Zambia) (2025) Compliance order for the management of tailing storage facility for the copper leach agitation processing plants. https://www.mmmd.gov.zm/wp-content/uploads/2025/06/Compliance-Order.pdf

[CR45] Ministry of Water and Sanitation (2025) Water declared safe following Sino-Metals pollution incident. Lusaka, Ministry of Water and Sanitation

[CR46] Mlambo V, Enaifoghe A, Mlambo D (2021) Examining the role of Africa in a changing global order. Afr J Public Aff 12(2):a3

[CR47] Musonda J, Larmer M (2023) Resource nationalism and political change. Mine nationalisation and the 2021 Zambian election. J South Afr Stud 49(3):397–414. 10.1080/03057070.2023.2265736

[CR48] Nassar NT, Lederer GW, Brainard JL, Padilla AJ, Lessard JD (2022) Rock-to-metal ratio: a foundational metric for understanding mine wastes. Environ Sci Technol 56(10):6710–6721 35467345 10.1021/acs.est.1c07875 PMC9118561

[CR49] Ndulo M (2013) Legal and regulatory frameworks for resource exploration and extraction. A global experience. In: Paper presented at the African Development Bank High-Level Policy Seminar on Optimizing the Benefits of Coal and Gas in Mozambique, Maputo

[CR50] Oliver-Smith A (1996) Anthropological research on hazards and disasters. Annu Rev Anthropol 25(1):303–328. 10.1146/annurev.anthro.25.1.303

[CR51] Oliver-Smith A (1999) What is a disaster? Anthropological perspectives on a persistent question. In: Oliver-Smith A, Hoffman S (eds) The Angry Earth. Disaster in Anthropological Perspective. New York, Routledge, pp. 18–34

[CR52] Oshionebo E (2021) Mineral mining in Africa. Legal and Fiscal Regimes. London, Routledge. 10.4324/9781351055543

[CR53] Owusu-Koranteng D (2008) Mining investment and community struggles. Rev Afr Political Econ 35(117):453–460. 10.1080/03056240802411115

[CR54] Petley D (2013) Global patterns of loss of life from landslides. Geol Today 29(3):116–120

[CR55] Petley D (2025) The 18 February 2025 Tailing Storage Facility Failure at Chambisi in Zambia, EOS, 24 February 2025. https://eos.org/thelandslideblog/chambishi-tsf-1

[CR56] Rana N, Ghahramani N, Evans SG, Small A, Skermer N, McDougall S, Take WA (2022) Global magnitude-frequency statistics of the failures and impacts of large water-retention dams and mine tailings impoundments. Earth Sci Rev 232:104144

[CR57] Sikamo J, Mwanza A, Mweemba C (2016) Copper mining in Zambia – history and future. J South Afr Inst Min Metall 116(6):491–496. 10.17159/2411-9717/2016/v116n6a1

[CR69] Sishekanu M, Katati M (2021) Subjectivity in the logic of Zambiaas environmental impact assessments (EIA) process: The bedrock of controversial EIA approvals. Law, Environ Develop J 17(1):40–54

[CR58] Solei K, Tinkler O (2016) Copper solvent extraction. Status, operating practices, and challenges in the African Copperbelt. J South Afr Inst Min Metall 116(6):553–560. 10.17159/2411-9717/2016/v116n6a10

[CR59] U.S. Embassy in Zambia (2025) Health alert – Acute heavy metal poisoning. Lusaka, U.S. Embassy. Available at: https://zm.usembassy.gov/health-alert-acute-heavy-metal-poisoning/

[CR60] United Nations Office for Disaster Risk Reduction (UNDRR) (2025) Collapse in Chambishi – Tailings failure is a canary in Zambia’s Copperbelt. Available at: https://www.undrr.org

[CR67] UN-Habitat (2026) Zambia. UN-Habitat. https://unhabitat.org/zambia?utm_source=chatgpt.com

[CR61] Wasilewski S, Skotniczny P (2015) Mining waste dumps – Modern monitoring of thermal and gas activities. Gospodarka Surowcami Miner 31(1):155–182. 10.1515/gospo-2015-0010

[CR62] World Bank (1992) Strategy for African Mining. World Bank, Washington, DC, (Technical Paper No. 181)

[CR63] World Bank (2002) Zambian copperbelt environmental project: environmental impact statement. Washington, DC

[CR64] Zadeh J (2025) Chinese Firm’s Zambia Mine Spill: $420 Million in Damages Sought, Discovery Alert, 2 September 2024. https://discoveryalert.com.au/zambia-mine-spill-2025-toxic-disaster/

[CR65] Zhao X, Gao X, Li D (2012) The stability analysis of Nantong coal mine waste dump, Chongqing and prevention measures. Appl Mech Mater 204–208:3526–3531

[CR66] Zhu X, Cui Y, Peng J, Jiang C, Guo W (2019) Erosion and transport mechanisms of mine waste along gullies. J Mt Sci 16(2):402–413. 10.1007/s11629-018-4981-7

